# Mace‐Like Plasmonic Au‐Pd Heterostructures Boost Near‐Infrared Photoimmunotherapy

**DOI:** 10.1002/advs.202204842

**Published:** 2023-01-04

**Authors:** Yanlin Feng, Xin Ning, Jianlin Wang, Zhaoyang Wen, Fangfang Cao, Qing You, Jianhua Zou, Xin Zhou, Teng Sun, Jimin Cao, Xiaoyuan Chen

**Affiliations:** ^1^ Key Laboratory of Cellular Physiology at Shanxi Medical University Ministry of Education and the Department of Physiology Shanxi Medical University Taiyuan 030001 China; ^2^ Departments of Diagnostic Radiology, Surgery, Chemical and Biomolecular Engineering and Biomedical Engineering Yong Loo Lin School of Medicine and Faculty of Engineering National University of Singapore Singapore 119074 Singapore; ^3^ Nanomedicine Translational Research Program NUS Center for Nanomedicine Yong Loo Lin School of Medicine National University of Singapore Singapore 117597 Singapore; ^4^ Clinical Imaging Research Centre Centre for Translational Medicine Yong Loo Lin School of Medicine National University of Singapore Singapore 117599 Singapore; ^5^ Institute of Molecular and Cell Biology Agency for Science, Technology, and Research (A*STAR) 61 Biopolis Drive, Proteos Singapore 138673 Singapore

**Keywords:** dendritic cells maturation, mace‐like gold‐palladium heterostructures, photoimmunotherapy, PD‐L1 blockade therapy, three negative breast cancer (TNBC)

## Abstract

Photoimmunotherapy, with spatiotemporal precision and noninvasive property, has provided a novel targeted therapeutic strategy for highly malignant triple‐negative breast cancer (TNBC). However, their therapeutic effect is severely restricted by the insufficient generation of tumor antigens and the weak activation of immune response, which is caused by the limited tissue penetration of light and complex immunosuppressive microenvironment. To improve the outcomes, herein, mace‐like plasmonic Au—Pd heterostructures (Au Pd HSs) have been fabricated to boost near‐infrared (NIR) photoimmunotherapy. The plasmonic Au Pd HSs exhibit strong photothermal and photodynamic effects under NIR light irradiation, effectively triggering immunogenic cell death (ICD) to activate the immune response. Meanwhile, the spiky surface of Au Pd HSs can also stimulate the maturation of DCs to present these antigens, amplifying the immune response. Ultimately, combining with anti‐programmed death‐ligand 1 (*α*‐PD‐L1) will further reverse the immunosuppressive microenvironment and enhance the infiltration of cytotoxic T lymphocytes (CTLs), not only eradicating primary TNBC but also completely inhibiting mimetic metastatic TNBC. Overall, the current study opens a new path for the treatment of TNBC through immunotherapy by integrating nanotopology and plasmonic performance.

## Graphical abstract

Mace‐like plasmonic Au—Pd heterostructures are designed to boost near‐infrared (NIR) photoimmunotherapy, by which Pd spikes on Au nanorod promotes hot electron generation and transfer, generating abundant heat and ROS to inhibit the primary tumor growth. Meanwhile, integration of physical immune activation, photoimmunotherapy, and *α*‐PD‐L1 can amplify the immune response and reverse the immunosuppressive microenvironment to eliminate the distant tumors.

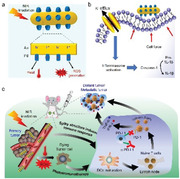

## 1 Introduction

Triple negative breast cancer (TNBC) has close ties to poor prognosis, high recurrence, high metastasis rate and mortality,^[^ ^1^ ^]^ and lacks treatment options. The ineffectiveness of traditional treatment of metastatic TNBC has led researchers to focus on cancer immunotherapy, which attacks cancer cells utilizing the patient's own immune system.^[^ ^2^, ^3^ ^]^ Despite the progress in clinical trials, only a small portion of patients benefit from immunotherapy because of insufficient activation of immune systems and autoimmune related adverse reactions.^[^ ^4^, ^5^ ^]^ Thus, intensive efforts have been made to enhance the immune responses for TNBC. Especially, photoimmunotherapy composed of photothermal therapy (PTT) or photodynamic therapy (PDT) and immunotherapy has been fascinating in promoting immune responses against tumors because of the excellent spatiotemporal controllability and noninvasive property.^[^ ^6^, ^7^, ^8^, ^9^, ^10^ ^]^ Specifically, activated by light, PTT and PDT agents could generate local heat and reactive oxygen species (ROS) damages in tumors, inducing immunogenic cell death (ICD). These dying tumor cells could release damage‐associated molecular patterns (DAMPs), such as calreticulin (CRT), which could act as an “eat me” signal to stimulate the antigen presenting function of dendritic cells (DCs).^[^ ^11^, ^12^, ^13^, ^14^ ^]^ Currently, researches have mostly focused on adjusting the light absorption of phototherapy agents to the near infrared (NIR) region to enhance the tissue penetration and trigger prominent ICD. Although promising, these DAMPs produced by phototherapy are not enough to activate a systematic immune response due to the negative immune regulation mechanisms. Therefore, it's urgent to develop a new photoimmune agent that integrates strong phototoxicity with inherent adjuvanticity to not only produce enough tumor antigen, but also effectively activate innate immunity for metastatic TNBCs therapy.

With unique electronic properties, plasma catalyst has emerged as a phototherapy platform to improve the phototherapeutic effect of cancer. Under incident light excitation, their localized surface plasmon resonance (LSPR) can enhance the electromagnetic field for energetic charge carrier generation.^[^ ^15^, ^16^ ^]^ Whereafter, these abundant charges go through the chemical and energy transformation process to generate ROS and through electron–phonon relaxation process to release vast heat.^[^ ^3^, ^17^, ^18^ ^]^ Furthermore, by finely tuning the anisotropic architecture, the charge carrier spatial separation could be further facilitated through the alignment of Fermi level on disparate metals,^[^ ^19^, ^20^, ^21^ ^]^ resulting in more abundant hot electron production for PTT and PDT. In addition, the LSPR of plasma catalyst can be facilely adjusted to the NIR region, providing deep tissue penetration. More importantly, the surface structures of plasma catalyst can be flexibly customized to better present antigens to DCs, since a spiky structure can activate and amplify the immune response through boosting the maturation of DCs.^[^ ^22^ ^]^

**Figure 1:**
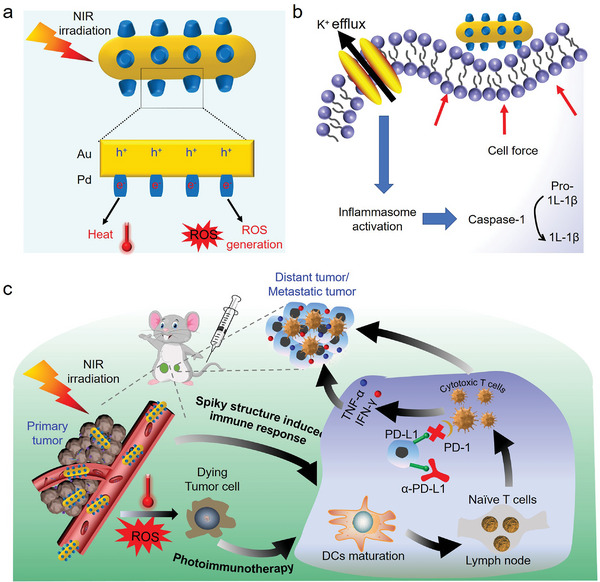
Schematic illustration of mace‐like plasmonic Au—Pd heterostructures boosting near‐infrared photoimmunotherapy for primary and distant or metastatic TNBC. a) NIR laser promotes the charge carrier spatial separation to release heat and ROS for PTT and PDT. b) The spiky surface of Au Pd HSs exerts mechanical stress on DCs, resulting in K^+^ efflux and inflammasome activation. c) Photoimmunotherapy of Au Pd HSs toward primary and distant or metastatic TNBC.

Herein, to achieve the significant immune response of phototherapy, we fabricated mace‐like Au—Pd heterostructures (Au Pd HSs) with enhanced phototoxicity and inherent adjuvanticity for TNBC therapy. Specifically, Pd spikes were orderly arranged on the surfaces of gold nanorods (Au NRs) via a scaled‐up strategy to form Au Pd HSs with an excellent plasmonic property and rough surface. With 808 nm laser irradiation, Au Pd HSs could largely generate hot electrons through amplifying the local electromagnetic field and decreasing the electron−phonon coupling.^[^ ^3^, ^23^, ^24^ ^]^ Immediately, these efficient hot electrons could promote heat release and ROS production, including singlet oxygen (^1^O_2_), superoxide radical (O_2_•^−^) and hydroxyl radical (•OH), affording outstanding PTT and PDT performance to increase immunogenicity (**Figure** **1**a). Simultaneously, the spiky surface of Au Pd HSs could exert mechanical stress to cause K^+^ efflux and activate the inflammasome of DCs, functioning as adjuvant to further maturate DCs and facilitate antigen presentation (Figure 1b,c). Then, these mature DCs would move to the tumor‐draining lymph nodes to activate the naive T cells and promote the infiltration of cytotoxic T lymphocytes (CTLs), eliminating both the primary and distant tumors. More importantly, Au Pd HSs could achieve outstanding photoimmune effects on metastatic tumors. Finally, to comprehensively activate the immune response, anti‐programmed death‐ligand 1 (*α*‐PD‐L1) was adopted to reverse the immunosuppressive T cells and increase CTLs to treat TNBC. In a word, Au Pd HSs could effectively activate the immune responses and inhibit the metastatic TNBC, providing new insight into immunotherapy by integrating nanotopology and plasmonic catalytic performance.

## 2 Results and Discussion

**Figure 2:**
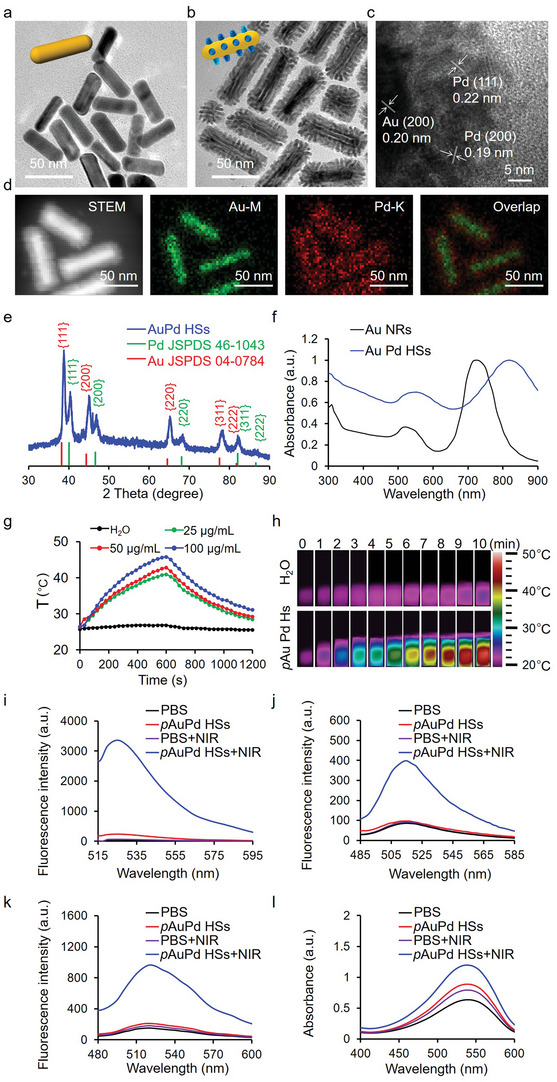
Physicochemical characterization of the synthesized Au Pd HSs and their property assessment. a,b) TEM images of Au NRs and Au Pd HSs. c) HRTEM image of Au Pd HSs. d) STEM and corresponding elemental mapping images of Au Pd HSs. e) The XRD pattern of Au Pd HSs. The corresponding peaks assigned by standard indexes of Au (red lines, JCPDS 04–0784) and Pd (green lines, JCPDS 46–1043). f) UV–Vis–NIR absorption spectra of Au Pd HSs. g) Heating and cooling curves of *p*Au Pd HSs (25, 50, and 100 µg mL^−1^) and water under 808 nm laser irradiation at a power density of 0.5 W cm^−2^. h) Infrared thermal images of *p*Au Pd HSs (100 µg mL^−1^). Total ROS, •OH, ^1^O_2_ and O_2_•^−^ assessments on *p*Au Pd HSs (100 µg mL^−1^) with or without 808 nm laser irradiation (0.5 W cm^−2^, 10 min) by i) DCF, j) APF, k) SOSG and l) superoxide anion assay, respectively. PBS was used as control.

Mace‐like Au Pd HSs was prepared through the anisotropic growth of Pd spikes on Au NRs surfaces. First, Au NRs were synthesized by seeded growth strategy.^[^ ^25^, ^26^ ^]^ Then, cetylpyridinium chloride (CPC) was introduced to direct the formation of Pd spikes on the surfaces of Au NRs. Typically, due to the special polarizability and compactness of the pyridinium headgroup, Pd precursors interacted with the pyridinium headgroup of CPC on Au NRs surfaces at 65 °C.^[^ ^27^ ^]^ Followed by the reduction agent of ascorbic acid (AA), small Pd domains attaching on the Au NRs were discerned, and grew over time in both number and length to form mace‐like Au Pd HSs. Transmission electron microscopy (TEM) images displayed that Au NRs with uniform sizes of 45.9 ± 7.7 nm in length and 10.9 ± 3.5 nm in diameter, while Au Pd HSs exhibited monodisperse mace‐like nanostructures, where Pd spiky shells homogeneously surrounded the inner Au NRs cores and the average length of Pd spike was 6.1 ± 1.9 nm (**Figure** **2**a,b). High resolution transmission electron microscopy (HRTEM) images (Figure 2c) revealed that the interplanar distances of 0.2 nm corresponded to the (200) plane of Au NRs core, and 0.19 and 0.22 nm matched with (200) and (111) lattice planes of Pd, respectively. The mace‐like Au Pd HSs structure and element distribution were further confirmed by scanning transmission electron microscope (STEM) image and corresponding X‐ray elemental mapping (Figure 2d). X‐ray diffraction (XRD) patterns (Figure 2e) clearly showed the diffraction peaks of Au (JCPDS 04–0784) and Pd (JCPDS 46–1043), further confirming the successful synthesis of highly crystalline Au Pd HSs without other impurity phase. UV–Vis–NIR absorption spectra indicated that Au Pd HSs redshifted the LSPR peak of Au NRs from 730 to 810 nm (Figure 2f).

To improve the biological compatibility for the potential biomedical application, the surface of Au Pd HSs was conjugated with thiol‐terminated PEG (PEG‐SH, *M*_w_ = 5000) to achieve PEG modified Au Pd HSs (*p*Au Pd HSs). The PEG content in Au Pd HSs was 6.86% as determined by thermogravimetric analysis (TGA) (Figure S1, Supporting Information). After PEGylation, the hydrodynamic sizes of Au Pd HSs in water and Dulbecco's Modified Eagle Medium (DMEM) increased from 141 ± 8.3 to 163.7 ± 11.7 nm (water) and 156.9 ± 8.5 to 194.2 ± 6.2 nm (DMEM), respectively. In addition, the zeta potentials of *p*Au Pd HSs changed from 0.3 ± 0.04 to −1.8 ± 0.3 eV (water) and −7.9 ± 0.7 to −14.3 ± 1.3 eV (DMEM), respectively. Moreover, after storage for 6 month (180 days), the absorption profile, the hydrodynamic size and zeta potential of *p*Au Pd HSs remained unchanged (Figure S2a–c, Supporting Information), indicating the excellent stability of *p*Au Pd HSs.

The photothermal performance of *p*Au Pd HSs was studied by monitoring the temperature elevation using thermometer and thermal infrared imaging with 808 nm laser irradiation (0.5 W cm^−2^, 10 min). Figure 2g,h shows that *p*Au Pd HSs induced significant temperature elevation with concentration‐dependent manner, and the photothermal conversion efficiency (*ƞ*) was further calculated to be 41.9% according to Roper's method (Figure 2g and Figure S3, Supporting Information).^[^ ^28^ ^]^ Moreover, the prominent photothermal performance of *p*Au Pd HSs was also laser power‐dependent (0.75 W cm^−2^, Figure S4, Supporting Information). The photodynamic performance under 808 nm laser irradiation was investigated by measuring the ROS of •OH, ^1^O_2_, and O_2_•^−^ generation ability. First, the total ROS generation ability was evaluated by 2′,7′‐dichlorodihydrofluorescein (DCF) assay. Figure 2i indicated that *p*Au Pd HSs exhibited significant DCF fluorescence enhancement under 808 nm laser irradiation, implying a large amount of ROS production. Then, the productions of •OH, ^1^O_2_ and •O_2_•^−^ were verified by 3′‐(p‐aminophenyl) fluorescein (APF), singlet oxygen sensor green (SOSG) and Micro Superoxide Anion Assay Kit. Figure 2j–l shows that *p*Au Pd HSs exhibited significant intensity of APF, SOSG fluorescence and superoxide anion absorbance with 808 nm laser irradiation, demonstrating the abundant generation of •OH, ^1^O_2_ and O_2_•^−^. In contrast, without NIR light irradiation, *p*Au Pd HSs displayed negligible generation of •OH, ^1^O_2_ or O_2_•^−^ species, indicating that the ROS is derived from the LSPR excitation. Moreover, the production of ROS is concentration dependent (Figure S5, Supporting Information), promising to effectively control cell death.

**Figure 3:**
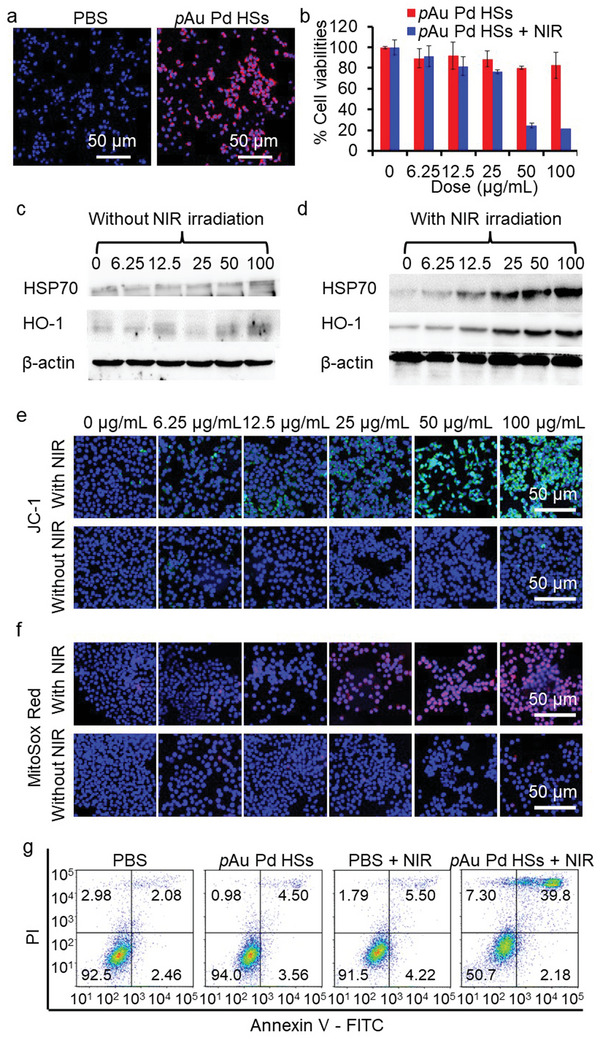
In vitro phototherapeutic effect of *p*Au Pd HSs in 4T1 cells. a) CLSM images of 4T1 cells treated with *p*Au Pd HSs‐RhB for 6 h, and 4′,6‐diamidino‐2‐phenylindole (DAPI) staining was used to visualize the nuclei. b) CCK‐8‐based cell viability assessment of 4T1 cells after treatment of *p*Au Pd HSs at various concentrations without or with 808 nm laser irradiation. c,d) Western blotting of HSP‐70 and HO‐1 expressions without or with NIR irradiation. CLSM images showing e) mitochondrial membrane depolarization (JC‐1, green) and f) superoxide generation (Mitosox Red, red). Cell nuclei were stained with DAPI (blue). g) Flow cytometric analysis of the apoptotic cells. For (b–f), 4T1 cells were treated with various concentrations of *p*Au Pd HSs for 24 h with and without 808 nm laser irradiation for 5 min at 0.5 W cm^−2^.

Encouraged by the notable photothermal and photodynamic effect of *p*Au Pd HSs, the potential phototherapeutic effects were assessed in murine mammary carcinoma (4T1) cells. RhB labeled *p*Au Pd HSs (*p*Au Pd HSs‐RhB) had efficient cell internalization in 4T1 cells after 6 h treatment as estimated by confocal fluorescence microscopy (CLSM) (**Figure** **3**a). Then, cell counting kit (CCK)‐8 assay was used to assess the biocompatibility of *p*Au Pd HSs. Figure 3b displays that *p*Au Pd HSs did not affect the cell viability, indicating the excellent biocompatibility. However, *p*Au Pd HSs induced significant decline in cell viability in a dose‐dependent manner with 808 nm laser irradiation. Meanwhile, calcein acetoxymethyl (AM)/propidium iodide (PI) live/dead cell staining assay (Figure S6, Supporting Information) further confirmed the PTT and PDT effect. Similar results could be also discovered in normal mouse embryonic fibroblast (NIH‐3T3) cells (Figure S7, Supporting Information) due to the inherent biosafety of *p*Au Pd HSs as well as the non‐specific toxicity of generated heat and ROS. However, the nanosized *p*Au Pd HSs could accumulate into the tumor site through enhanced permeability and retention (EPR) effect and produce specific phototoxicity with tumors under irradiation, attributing to the short life times of ROS and limited transfer of heat. Thus, *p*Au Pd HSs is a promising photoimmunotherapy agent for TNBC with high efficiency and negligible side effects.

Generally, PTT‐induced cellular hyperthermia could trigger heat shock protein (HSP) expression,^[^ ^29^, ^30^ ^]^ while PDT‐induced cellular ROS production could prompt phase II enzyme expression (typically heme oxygenase 1 [HO‐1]).^[^ ^31^, ^32^ ^]^ Thus, to verify that *p*Au Pd HSs induced cell death under NIR laser irradiation was caused by PTT‐induced cellular hyperthermia and PDT‐induced cellular ROS production, HSP70 and HO‐1 protein expressions in 4T1 cells were analyzed using western blot. Figure 3c,d implies that with 808 nm laser irradiation, the expression of HSP70 and HO‐1 was upregulated in a dose‐dependent manner, while *p*Au Pd HSs alone without laser irradiation was unable to induce HSP70 or HO‐1 expression. Elevated expressions of HSP70 and HO‐1 would activate cellular oxidative stress signaling pathways, leading to cellular ROS production and mitochondrial dysfunction.^[^ ^31^ ^]^ DCF assay was used to assess the cellular ROS level. Figure S8, Supporting Information, shows that *p*Au Pd HSs could induce dose‐dependent DCF fluorescence enhancement with 808 nm laser irradiation, while *p*Au Pd HSs or NIR alone could not enhance the DCF fluorescence. Mitochondrial dysfunction, including mitochondrial membrane depolarization and mitochondrial superoxide production, was examined by fluorescent staining of JC‐1 and MitoSox Red. Confocal fluorescence microscopy (CLSM) images demonstrated that *p*Au Pd HSs significantly increased the intensities of JC‐1 green fluorescence and the Mitosox Red fluorescence when triggered by 808 nm laser irradiation, indicating mitochondrial membrane potential depolarization and superoxide generation. In contrast, *p*Au Pd HSs or NIR alone could not induce mitochondrial dysfunctions (Figure 3e,f). Mitochondrial dysfunction‐induced apoptosis is another important marker for oxidative stress injury. Annexin V‐FITC (AV)/propidium iodide (PI) apoptosis assay in Figure 3g indicated that *p*Au Pd HSs or NIR alone did not induce noticeable 4T1 cell apoptosis, while *p*Au Pd HSs with NIR laser irradiation induced 41.98% apoptosis. In a word, *p*Au Pd HSs with NIR irradiation exhibited a significant destructive effect on the tumor cells, and this therapeutic action was likely attributed to the activation of hierarchical oxidative stress.

**Figure 4:**
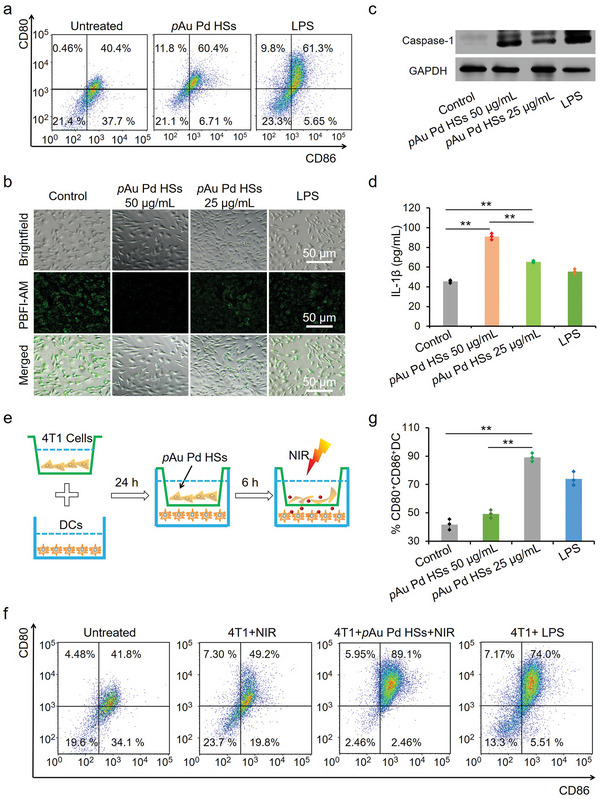
In vitro DCs maturation effect of *p*Au Pd HSs. a) Flow cytometry analysis of CD80 and CD86 expressions on DCs after co‐culture with *p*Au Pd HSs (50 and 25 µg mL^−1^). LPS (100 ng mL^−1^) was used as the positive control. b) Intracellular K^+^ detection after cells were treated with *p*Au Pd HSs for 24 h. c) Western blot analysis of Caspase‐1 expression. d) IL‐1*β* secretion detected by ELISA assay. e) Scheme showing the transwell co‐culture system. f) Flow cytometry analysis of CD80 and CD86 expressions on BMDCs after co‐culture with *p*Au Pd HSs (50 and 25 µg mL^−1^) under 808 nm laser irradiation. g) Quantification of CD80 and CD86 expression of (f). Data are expressed as means ± s.d. (*n* = 3). Statistical significances were calculated via Student's *t* test. **p < 0.01.

A few studies have reported that nanopillar structures or nanopatterned features could influence immune cells, especially DCs.^[^ ^33^, ^34^, ^35^, ^36^, ^37^, ^38^ ^]^ Co‐stimulatory molecules (CD80 and CD86) expressions have been used to estimate the maturation level of DCs. Flow cytometry analysis in **Figure** **4**a shows that *p*Au Pd HSs could increase the percentage of matured DCs by 20%, like the positive control (lipopolysaccharide [LPS]) of 20.9%, which is a powerful promoter of the maturation of DC.^[^ ^39^, ^40^ ^]^ The DCs maturation may be ascribed to the potassium (K^+^) efflux and inflammasome activation. K^+^‐sensitive fluorophore fluorescence (PBFI‐AM)^[^ ^41^ ^]^ was significantly inhibited by *p*Au Pd HSs (Figure 4b). ELISA assessments of IL‐1*β* levels and Western blot analysis of Caspase‐1 expression^[^ ^42^ ^]^ implied that *p*Au Pd HSs could significantly elevate the IL‐1*β* levels and Caspase‐1, which are the biomarkers of inflammasome (Figure 4c,d). All the above results confirmed that *p*Au Pd HSs alone could maturate DCs through the K^+^ efflux and inflammasome activation.

Besides the physical stimulation, tumor cell residues after phototherapy could act as tumor‐associated antigens to promote DCs maturation and finally induce antitumor immune responses.^[^ ^43^, ^44^ ^]^ A transwell co‐culture system was used to mimic tumors in vivo. In the transwell system, 4T1 cells were seeded in the upper chamber while DCs were added in the lower chamber of the transwell. The 4T1 cell residues were generated by treating with *p*Au Pd HSs and NIR irradiation to mimic the ablation of tumors in vivo. Only the tumor antigens of 4T1 cells or 4T1 cell residues were allowed to pass the membrane through the micropores to stimulate DCs (Figure 4e). Then, DCs were stained with CD11c, CD80, and CD86 for flow cytometry analysis. Figure 4f,g displayed that *p*Au Pd HSs could remarkably maturate DCs with NIR light irradiation. As a result, we concluded that integrating nanotopology and phototherapy could significantly enhance the immune response mainly through maturation of DCs.

**Figure 5:**
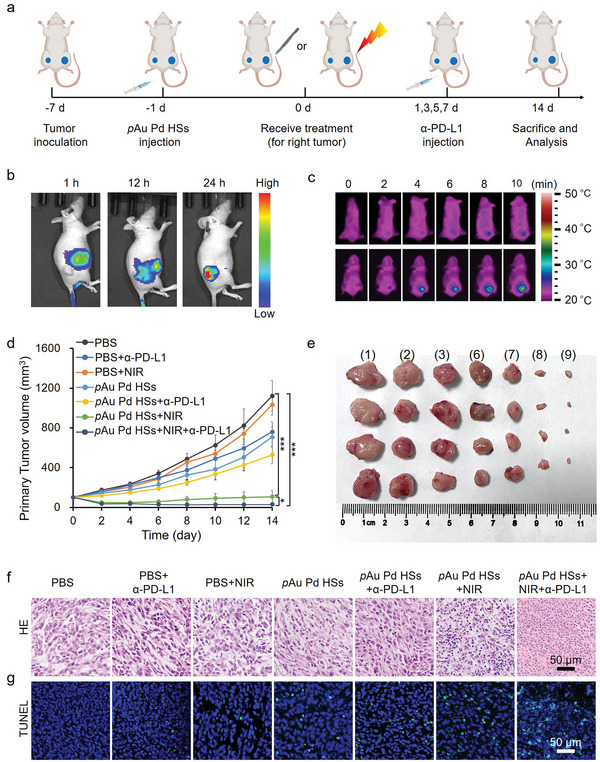
Anti‐tumor effect of *p*Au Pd HSs. a) Schematic illustration of the therapeutic protocol in 4T1 tumor mice. b) Fluorescence images of mice at 1, 12, and 24 h after intravenous injection of *p*Au Pd HSs‐Cy7. c) Infrared thermal images of mice treated with intravenous PBS or *p*Au Pd HSs at 24 h post‐injection with 808 nm laser radiation (0.5 W cm^−2^, 10 min). d) Tumor growth curves of seven groups after different treatments as indicated (*n* = 4/group). e) Representative photos of primary tumors dissected from seven groups of mice at the end of treatment. i) PBS; ii) PBS+*α*‐PD‐L1; iii) PBS+NIR; vi) *p*Au Pd HSs; vii) *p*Au Pd HSs+*α*‐PD‐L1; viii) *p*Au Pd HSs+NIR; ix) *p*Au Pd HSs+NIR+*α*‐PD‐L1. f) H&E and g) TUNEL staining images of the primary tumor. *p < 0.05, ** *p* < 0.01, *** *p* < 0.001.

Inspired by the satisfactory in vitro phototherapeutic performance, as well as the integrated immunoadjuvant property of *p*Au Pd HSs on 4T1 cells, in vivo therapeutic effects of *p*Au Pd HSs were further investigated on TNBC using a 4T1 tumor model. The animal experiment is designed in **Figure** **5**a. The right and left flanks of each BALB/c mouse were inoculated with 2 × 10^6^ and 1 × 10^6^ 4T1 cells to mimic the primary and distant tumors, respectively. When the primary tumor volume reached about 100 mm^3^, the mice were randomized into 9 groups (4 mice in each group): 1) PBS; 2) PBS+*α*‐PD‐L1; 3) PBS+NIR; 4) surgery; 5) surgery+*α*‐PD‐L1; 6) *p*Au Pd HSs; 7) *p*Au Pd HSs +*α*‐PD‐L1; 8) *p*Au Pd HSs+NIR; and 9) *p*Au Pd HSs+NIR+*α*‐PD‐L1. For groups (3), (8) and (9), the primary tumor was irradiated with 808 nm laser for 5 min at 0.5 W cm^−2^. For group (4) and (5), the primary tumors of mice were ablated by surgery. Afterward, mice of groups (2), (5), (7) and (9) were intravenously (i.v.) injected with *α*‐PD‐L1 antibody of 20 µg per mouse on day 1, 3, 5 and 7. The tumor sizes and body weights of the mice in different groups were measured every two days. First, fluorescence imaging together with inductively coupled plasma‐optical emission spectroscopy (ICP‐OES) analysis were performed to track the biodistribution of *p*Au Pd HSs. Cy7‐labeled *p*Au Pd HSs (*p*Au Pd HSs‐Cy7) was intravenously injected and the fluorescence images were acquired at 1, 12, and 24 h post‐administration. Figure 5b shows noticeable accumulation of *p*Au Pd HSs in tumor at 24 h post‐injection, proving that *p*Au Pd HSs could passively target to the tumor tissue. The enhanced permeability and retention (EPR) effect can also be proved by the distribution assay (Figure S9a, Supporting Information), which displayed that *p*Au Pd HSs mainly accumulated in the liver, spleen, tumor and lung at 24 h post‐injection. Although small amounts of *p*Au Pd HSs accumulated in the kidney, they could be can be excreted out through renal pathway, which is suggested by the examined Au content in urine at extended time points after i.v. injection (Figure S9b, Supporting Information).

The in vivo PTT effect of *p*Au Pd HSs was evaluated by monitoring the temperature change in the tumor region of mice. The infrared thermal image (Figure 5c) showed that the tumor temperature increased from 25.1 °C to about 42.8 °C with NIR laser irradiation after intravenous administration of *p*Au Pd HSs. Tumor growth curves revealed that *p*Au Pd HSs+NIR and *p*Au Pd HSs+NIR+*α*‐PD‐L1 groups effectively inhibited the primary tumor growth (Figure 5d), while *α*‐PD‐L1 injection alone only partially delayed tumor growth in the early stage. Surprisingly, *p*Au Pd HSs inhibited the tumor growth to some extent, probably due to the immune activation ability of *p*Au Pd HSs. The representative photos of tumor tissues at the end of treatment in Figure 5e also verified the inhibition effect. Tumor tissue sections stained with hematoxylin and eosin (H&E) and terminal deoxynucleotidyl transferase dUTP nick‐end labeling (TUNEL) corroborated that *p*Au Pd HSs+NIR+*α*‐PD‐L1 triggered the most severe damage to the primary tumor tissues, followed by *p*Au Pd HSs+NIR and *p*Au Pd HSs, while little cell necrosis was observed in other groups (Figure 5f,g).

**Figure 6:**
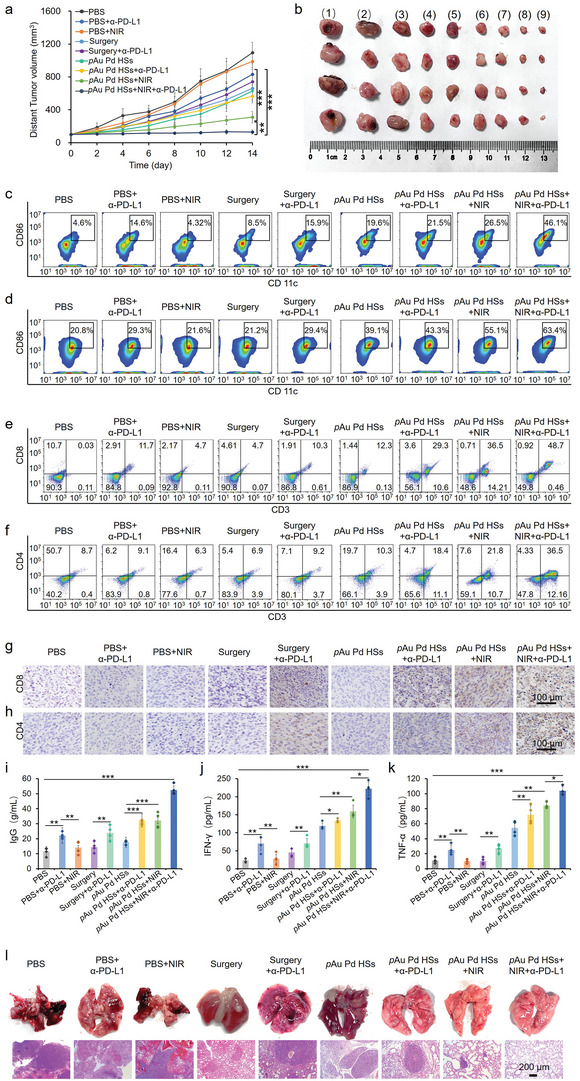
Anti‐tumor effect of *p*Au Pd HSs of distant tumors. a) Tumor growth curves of nine groups after different treatments (*n* = 4/group). b) Representative photos of distant tumors dissected from nine groups of mice at the end of treatment. i) PBS; ii) PBS+*α*‐PD‐L1; iii) PBS+NIR; iv) surgery; v) surgery+*α*‐PD‐L1; vi) *p*Au Pd HSs; vii) *p*Au Pd HSs+*α*‐PD‐L1; viii) *p*Au Pd HSs+NIR; ix) *p*Au Pd HSs+NIR+*α*‐PD‐L1. Typical flow cytometry plots of CD86 expressions in c) the distant tumor tissue and d) lymph nodes after different treatments. Typical flow cytometry plots of e) CD8^+^ T cells and f) CD4^+^ T cells as well as immunohistochemical (IHC) staining for g) CD8^+^ T cells and h) CD4^+^ T cells in the distant tumors after different treatments. i) Serum IgG, j) IFN‐*γ* and k) TNF‐*α* levels of nine groups after different treatments detected by ELISA. l) Representative lung photograph and corresponding H&E staining of the lung tissues. Data are expressed as means ± s.d. (*n* = 4). Statistical significances were calculated via Student's *t* test. *p < 0.05, ** *p* < 0.01, *** *p* < 0.001.

Then, the growth profile of the distant tumors was also investigated. **Figure** **6**a,b shows the growth curves and the representative photos of the distant tumors. It was also found that tumor growth was partially delayed only in the early stages with *α*‐PD‐L1 injection alone, while the *p*Au Pd HSs+NIR group showed a better inhibition effect. Tumor growth was obviously inhibited in the *p*Au Pd HSs+NIR+*α*‐PD‐L1 group, which suggested not only a synergistic therapeutic effect of *p*Au Pd HSs, NIR irradiation and *α*‐PD‐L1 in strengthening immunity systemically, but also an inspiring abscopal effect. Meanwhile, no significant reduction in body weight was observed in any of the groups except for the surgery groups (Figure S10, Supporting Information), indicating the excellent biocompatibility of *p*Au Pd HSs. Meanwhile, no obvious abnormality or damage was observed in the heart, liver, spleen, lung, and kidneys (Figure S11, Supporting Information). H&E and TUNEL staining demonstrated that the distant tumor tissues suffered from severe cell damage in the *p*Au Pd HSs+NIR+*α*‐PD‐L1 group, while little cell necrosis was observed in the other groups (Figure S12, Supporting Information), suggesting the effectiveness and biosafety of photoimmunotherapy.

Notably, the suppression of distant tumors in mice of the *p*Au Pd HSs+NIR+*α*‐PD‐L1 group could be attributed to the activation of systemic antitumor immune responses. Thus, to further disclose the immune mechanism in vivo, the maturation of DCs in the tumor tissue and lymph nodes was first evaluated after 4 days of different treatments. Obviously, enhanced DC maturation was observed both in the distant tumor tissue (Figure 6c) and lymph nodes (Figure 6d) after being treated with *p*Au Pd HSs+NIR+*α*‐PD‐L1 group. DC maturation was conducive to the initiation of T‐cell‐mediated immune responses, in which cytotoxic T cells (CD8^+^ and CD4^+^ T cells) will directly attack cancer cells to trigger the apoptosis.^[^ ^45^, ^46^ ^]^ As a result, CD8^+^ and CD4^+^ T cells in CD3^+^ T cells in the primary and distant tumors were analyzed. Flow cytometry analysis displayed that CD8^+^ and CD4^+^ T cells were remarkably enhanced both in the primary (Figure S13, Supporting Information) and distant tumors (Figure 6e,f and Figure S14, Supporting Information), suggesting the significant antitumor immune responses induced by *p*Au Pd HSs combined with *α*‐PD‐L1 under 808 nm irradiation. Because CD4 is also expressed by the regulatory T (Treg) cells, which could suppress the antitumor effect,^[^ ^47^ ^]^ Treg cells (CD3^+^CD4^+^CD25^+^) in the left tumors were further analyzed. Figure S15, Supporting Information, demonstrates that the *p*Au Pd HSs+NIR+*α*‐PD‐L1 group could effectively inhibit the Treg cells, indicating that the antitumor immunity was contributed from the T cells recruited without the risk of Treg cells. The infiltrations of CD8^+^ and CD4^+^ T cells were further examined by immunohistochemistry (Figure 6g,h) and immunofluorescence (Figure S16, Supporting Information). It was found that the *p*Au Pd HSs+NIR+*α*‐PD‐L1 group showed the most significantly expanded CD8^+^ T cells and CD4^+^ T cells, implying effective antitumor immune response activation. Moreover, the serum immune globulin G (IgG) content of different groups was tested since IgG plays an important role in immune effector cells.^[^ ^48^ ^]^ Figure 6i reveals that *p*Au Pd HSs alone could elevate the level of IgG, and this performance could be further enhanced with NIR laser irradiation. Finally, *p*Au Pd HSs+NIR+*α*‐PD‐L1 group showed the highest IgG generation. Meanwhile, serum tumor necrosis factor *α* (TNF‐*α*) and interferon *γ* (IFN‐*γ*) levels were analyzed because TNF‐*α* can effectively indicate the anti‐tumor immune responses^[^ ^49^ ^]^ and IFN‐*γ* plays a key role in intracellular immunity against cancer.^[^ ^50^ ^]^ Figures 6j and 6k displayed that IFN‐*γ* and TNF‐*α* levels were obviously increased in the *p*Au Pd HSs group, which could be further raised by NIR laser and *α*‐PD‐L1, respectively. Moreover, *p*Au Pd HSs+NIR+*α*‐PD‐L1 group demonstrated the strongest inhibitory effect on the lung metastasis as verified by the representative lung photograph and corresponding H&E staining of the lung tissues (Figure 6l). In general, the synergistic anti‐tumor effect was ascribed from the physical immune stimuli of the spiky structure and tumor‐associated antigens generation after photo destruction of primary tumors generated. Then, the infiltrating cytotoxic CD8^+^ T cells and increased TNF‐*α* and IFN‐*γ* levels could infiltrate and kill the remaining tumor cells in distant tumors. Furthermore, the immune suppression of T cells was effectively relieved, insuring the quantity of T cells in distant tumors after *α*‐PD‐L1 treatment. All the above immune responses resulted in the efficacious abscopal anti‐tumor effect.

## 3 Conclusion

In summary, we have synthesized mace‐like plasmonic Au Pd HSs to boost NIR photoimmunotherapy. Pd spikes on Au nanorod not only successfully regulated the LSPR to the NIR region, but also largely promoted hot electron generation and transfer, demonstrating outstanding NIR PTT and PDT. Meanwhile, the spiky surface also promoted the maturation and activation of DCs to amplify the immune response. Furthermore, Au Pd HSs effectively alleviated immune tolerance and promoted intratumoral infiltration of CTLs by combining with the clinically approved *α*‐PD‐L1. Finally, CTLs promoted the cytokines secretion to assist cancer immunotherapy. Collectively, the remarkable synergistic effects in eliminating primary and distant tumors confirmed the excellent immune activation effect of Au Pd HSs. Above all, this work demonstrated the great potential of integrating physical immune activation and photoimmunotherapy, offering a new strategy for fighting TNBC clinically.

## 4 Experimental Section

### Synthesis of Au NRs

In order to synthesize Au NRs, a seed‐mediated growth method was used according to the literature.^[^ ^26^ ^]^ First, HAuCl_4_ solution (48.56 mm, 60 µL) was mixed with CTAB solution (0.1 m, 10 mL), and then the freshly prepared NaBH_4_ solution (0.01 m, 0.6 mL) in ice water was quickly injected under vigorous stirring. After stirring for 2 min, the brown‐yellow solution was aged at 30 °C for 2 h. To prepare the growth solution, 1.232 g CTAC and 0.308 g NaOL were dissolved in 100 mL warm water (50–70 °C) for 150 min under stirring. Subsequently, 9.6 mL AgNO_3_ solution (4 mm) was added, vigorously stirred for 30 s and let set for 15 min. After the static reaction, 100 mL HAuCl_4_ (1 mm) solution was added and stirred slowly for 90 min. Then 1.92 mL HCl was added and stirred for 15 min. Afterward, 0.5 mL AA (64 mm) was added and stirred vigorously for 30 s. Finally, 0.5 mL of the prepared seed solution was quickly injected into the growth solution and stirred for 1 min, and let stand at 30 °C overnight for growth. The resultant solution was centrifugated at 10 000 rpm for 10 min and washed with pure water for 2 times and redispersed in 24 mL of water.

### Synthesis of Au Pd HSs

Au Pd HSs were prepared according to the literature with slight modification.^[^ ^24^, ^51^ ^]^ Briefly, 12 mL Na_2_PdCl_4_ (10 mm) and 24 mL prepared Au NRs were added to 240 mL CPC (10 mm) solution at 65 °C. Then, 4.8 mL of freshly prepared AA solution (100 mm) was injected and stirred vigorously for 2 min. After being undisturbed for 2 h at 65 °C, the resulting solution was centrifuged at 10 000 rpm for 10 min and washed with pure water for 2 times to achieve Au Pd HSs.

### Surface Modification of Au Pd HSs

mPEG‐SH solution (6 mg mL^−1^, 10 mL) aqueous solution was mixed with Au Pd HSs (6 mg mL^−1^, 10 mL) under stirring for 24 h at room temperature. The additional mPEG‐SH was removed by centrifugated at 10 000 rpm for 10 min, washed with pure water for 2 times, and obtained by freeze‐drying.

### RhB Labeling of pAu Pd HSs (pAu Pd HSs‐RhB)

RhB was first reacted with NH_2_‐PEG‐SH (*M*_w_ = 5000). Briefly, 1 mg of NH_2_‐PEG‐SH was dissolved in 0.5 mL of 0.1 m sodium bicarbonate buffer (pH = 8.3), followed by the addition of 5 µL of 10 mm RhB stock solution dropwise. After being stirred at room temperature for 24 h, 1 mL of *p*Au Pd HSs aqueous suspension (1 mg mL^−1^) was added. The solution was vortexed immediately and then incubated at 4 °C for overnight, followed by centrifuging at 10 000 rpm for 10 min and washed with water three times to achieve *p*Au Pd HSs‐RhB.

### Photothermal Measurement and Infrared Thermal Imaging of pAu Pd HSs

1 mL *p*Au Pd HSs (100 µg mL^−1^) was placed in a quartz colorimeter and irradiated with 808 nm laser at 0.5 W cm^−2^ for 10 min. After that, the laser was removed and the system was cooled naturally for 10 min. Temperature values were recorded every 30 s for drawing photothermal curves. Infrared thermal images of *p*Au Pd HSs were obtained from Infrared Thermal Imager (Fotric 1104) every minute.

### Photodynamic Performance of pAu Pd HSs

The total ROS, hydroxyl radical (•OH), singlet oxygen (^1^O_2_) and superoxide anion (•O_2_^−^) generation of *p*Au Pd HSs were determined by DCF, APF, SOSG and Micro Superoxide Anion Assay Kit, respectively. 80 µL of DCF (29 µm), APF (10 µm) and SOSG (12 µm) solution was added to each well of the 96‐well plate, then 20 µL *p*Au Pd HSs was added to each well. The above solution was irradiated with 808 nm laser (0.5 W cm^−2^) for 10 min. After incubation for 24 h, DCF, APF or SOSG fluorescence emission spectra were collected with the excitation wavelength of 490, 470 or 394 nm. •O_2•_^−^ was detected by Micro Superoxide Anion Assay Kit according to manufacturer’ protocol.

### Cell Uptake of pAu Pd HSs

4 × 10^4^ 4T1 cells were seeded in each well of 24‐well plate for overnight growth. After the cells were treated with PBS or *p*Au Pd HSs‐Cy7 (20 µg mL^−1^) for 6 h, the cells were washed with PBS for three times and stained with 1 µm DAPI for fluorescence images captured with a fluorescence microscope (Olympus, Tokyo, Japan).

### In Vitro Phototherapeutic Effect

4T1 cells were inoculated into 96‐well plates at a cell density of 10^4^ per well for overnight growth. The culture medium was exchanged by 100 µL of fresh medium containing 0–100 µg mL^−1^ *p*Au Pd HSs. After 6 h of incubation, the cells were irradiated with 808 nm laser (0.5 W cm^−2^) for 10 min and cultured for another 18 h. The control group was incubated at 37 °C for 24 h under dark conditions. Cell counting kit (CCK)‐8 assay was used to detect the cytotoxicity. For live/dead cell staining, cells were washed with PBS for three times and stained with calcein AM (2 µm) and propidium iodide (PI, 4 µm) for 0.5 h and visualized with fluorescence microscope (Olympus; Tokyo, Japan).

### Intracellular ROS Detection

4T1 cells were inoculated into 12‐well plates at a cell density of 8 × 10^4^ per well for overnight growth. The culture medium was exchanged with 800 µL of fresh medium containing 0–100 µg mL^−1^ *p*Au Pd HSs. After 6 h of incubation, the cells were irradiated with 808 nm laser (0.5 W cm^−2^) for 10 min and cultured for another 16 h. The control group without NIR laser irradiation was incubated at 37 °C for 22 h under dark conditions. H2DCFDA (10 µm) were added to each well for another 2 h. The cells were washed twice with PBS and visualized with fluorescence microscope (Olympus; Tokyo, Japan).

### Western Blot Analysis of HO‐1, HSP70, and Caspase‐1 Protein Expression

4T1 cells were inoculated into 6‐well plates at a cell density of 1.6 × 10^5^ per well for overnight growth. The culture medium was exchanged with 1.6 mL of fresh medium containing 0–100 µg mL^−1^ *p*Au Pd HSs. After 6 h of incubation, the cells were irradiated with 808 nm laser (0.5 W cm^−2^) for 10 min and cultured for another 18 h. The control group without NIR laser irradiation was incubated at 37 °C for 24 h under dark conditions. The cells were lysed and collected with a cell lysis buffer and centrifuged to measure the protein content in the supernatant using the Bradford method. By calculation, the protein in each sample was quantified to 20 µg and 10% SDS‐PAGE gel was prepared for electrophoresis and transferred to a PVDF membrane. After sealing, *β*‐actin (1:1000, Beyotime), HSP70 antibody (1:1000, abcam), Caspase‐1 antibody (1:1000, Proteintech), or HO‐1 monoclonal antibody (1:1000, abcam) was co‐incubated with membranes for 12 h. After being washed with TBST solution, goat anti‐mouse horseradish peroxidase‐conjugated secondary antibody (1:1000, Beyotime) was added to incubate with the membrane for 1 h. After being washed 3 times with TBST buffer, the membrane was visualized by Bio‐Rad imaging system.

### Detection of Mitochondrial Membrane Potential and Superoxide Generation

Mitochondrial membrane potential and superoxide production were detected by JC‐1 and Mitosox Red fluorescent dyes. 4T1 cells were inoculated into 12‐well plates at a cell density of 8 × 10^4^ per well for overnight growth. The culture medium was exchanged with 800 µL of fresh medium containing 0–100 µg mL^−1^ *p*Au Pd HSs. After 6 h of incubation, the cells were irradiated with 808 nm laser (0.5 W cm^−2^) for 10 min and cultured for another 18 h. The control group without NIR laser irradiation was incubated at 37 °C for 24 h under dark conditions. The cells were washed with PBS two times and stained with 5 µm JC‐1 or Mitosox Red dye for 15 min. The nuclei were stained with 5 µm Hoechst. Finally, the cells were washed twice with PBS and visualized using fluorescence microscope (Olympus; Tokyo, Japan).

### Cell Apoptosis Measurement Analyzed by Flow Cytometry

4T1 cells were inoculated into 6‐well plates at a cell density of 1.6 × 10^5^ per well for overnight growth. The culture medium was exchanged with 1.6 mL of fresh medium containing 50 µg mL^−1^ *p*Au Pd HSs. After 6 h of incubation, the cells were irradiated with 808 nm laser (0.5 W cm^−2^) for 10 min and cultured for another 18 h. The control group without NIR laser irradiation was incubated at 37 °C for 24 h under dark conditions. The cells were digested with trypsin free of EDTA, washed twice with PBS, stained with Annexin V‐FITC/PI kit, and then detected by flow cytometry (BD AccuriC6 Plus).

### Tumor Xenograft

Female BALB/c mice aged 4–6 weeks old were bought from Vital River (Beijing) and used as xenograft mouse models. The procedures involving experimental animals were in accordance with protocols approved by the Animal Research Ethics Committee of the Shanxi Medical University. 2 × 10^6^ and 1 × 10^6^ 4T1 cells were injected into the right and left subcutaneous tissues of BALB/C mice to simulate primary and distant metastatic tumors.

### Preparation of DCs and In Vitro Stimulation of DCs by pAu Pd HSs

Dendritic cells were obtained from bone marrow from clean grade healthy BALB/c mice approximately 6‐weeks‐old. The mice were sacrificed by dislocation, and the femur and tibia of the hind limb of the mice were separated aseptically, soaked in 75% alcohol for 5 min, and washed in PBS twice. The two ends of the bone were cut off, and the RMI1640 liquid was extracted with a 5 mL sterile syringe into the bone marrow cavity to be rinsed out repeatedly until the bone marrow cavity turned white. Bone marrow suspension was filtered by 200 mesh, centrifuged at 2000 rpm for 10 min, and the supernatant was discarded. The cell precipitates were suspended in RPMI‐1640 medium in 24‐well culture plates and the concentration was adjusted to 5 × 10^9^ cells/L. After incubation for 4 h, the cells adhesion particles were removed, replaced with fresh culture and GM‐CSF (20 ng mL^−1^) and IL‐4 (10 ng mL^−1^) was added. The morphological changes of cells were observed with a microscope every day, and half the amount of liquid was changed every other day. Suspended cells were collected after incubation for 6 days to obtain DCs.

To study the mace like *p*Au Pd HSs‐mediated promotion of DCs maturation, acquired DCs above were inoculated into 6‐well plates and 1.6 mL of fresh medium was added, containing 25 and 50 µg mL^−1^ *p*Au Pd HSs or LPS (100 ng mL^−1^). After 24 h, the DCs were collected and incubated with anti‐mouse CD16/32 to reduce non‐specific binding to FCR. After that, DCs were stained with anti‐mouse CD11c FITC, anti‐mouse CD86 PE, and anti‐mouse CD80 APC for flow cytometry. FITC anti‐mouse Rat lgG2b *κ* isotype antibody, PE anti‐mouse Rat lgG2b *κ* isotype antibody, and APC anti‐mouse Rat lgG1 *κ* isotype antibody were also used as isotype controls.

To study the DCs maturation effect of the tumor‐associated antigens from tumor cell residues after phototherapy, DCs‐4T1 coculture transwell system was applied. 4T1 cells were seeded in the upper chamber while DCs were added in the lower chamber of the transwell (6 well‐plate, 0.4 µm sized microporous membrane) for overnight growth. Then, fresh medium containing 50 µg mL^−1^ *p*Au Pd HSs or LPS (100 ng mL^−1^) was added upper chamber. After being co‐incubated with 4T1 cell for 6 h, 4T1 cells were irradiated with 808 nm laser (0.5 W cm^−2^) for 10 min and cultured for another 18 h. The control group without NIR laser irradiation was incubated at 37 °C for 24 h under dark conditions. Supernatant of 4T1 cells or fragments of 4T1 cells and tumor antigens could cross the membrane to stimulate DCs. The maturation of DCs was analyzed by flow cytometry as the above protocol.

### Fluorescence Microscopy for K^+^ Efflux Analysis

DCs were inoculated into 12‐well plates at a cell density of 8 × 10^4^ per well, after overnight growth at 37 °C and 5% CO_2_. The culture medium was removed and 0.8 mL of fresh medium was added, containing 0–100 µg mL^−1^ *p*Au Pd HSs and LPS (100 ng mL^−1^). After 24 h of incubation, the culture medium was removed and the cells were washed twice with PBS, 400 µL medium was added to the wells, and 10 mm PBFI‐AM and 10 mm Pluronic F‐127 were included per well. After 2 h of incubation, the cells were washed twice with PBS and visualized using fluorescence microscope (Olympus; Tokyo, Japan).

### In Vivo Biodistribution Analysis

When the tumors reached about 100 mm^3^, the mice were intravenously injected with 200 µL *p*Au Pd HSs (15 mg kg^−1^ mouse). At the end of the treatment, major organs were collected for Au element based inductively coupled plasma‐optical emission spectroscopy (ICP‐OES) analysis.

### In Vivo Infrared Thermal Imaging

When the tumors reached around 100 mm^3^, the mice were intravenously injected with 100 µL of *p*Au Pd HSs. At 24 h postinjection, the tumors were irradiated with 808 nm laser (0.5 W cm^−2^, 5 min). The infrared thermal images were obtained by Fotric 1204 every 2 min.

### In Vivo Immunophototherapy of pAu Pd HSs Synergized with *α*‐PD‐L1 for Primary and Distant Tumors Under NIR Irradiation

The experiment was started when the primary tumor volume reached about 100 mm^3^, mice were randomly divided into 9 groups (4 mice in each group): 1) PBS; 2) PBS+*α*‐PD‐L1; 3) PBS+NIR; 4) surgery; 5) surgery+*α*‐PD‐L1; 6) *p*Au Pd HSs; 7) *p*Au Pd HSs +*α*‐PD‐L1; 8) *p*Au Pd HSs+NIR; and 9) *p*Au Pd HSs+NIR+*α*‐PD‐L1. For groups (3), (8) and (9), the primary tumor was irradiated with 808 nm laser for 5 min at 0.5 W cm^−2^. For group (4) and (5), the primary tumors of mice were ablated by surgery. Afterward, mice of groups (2), (5), (7) and (9) were intravenously (i.v.) injected with *α*‐PD‐L1 antibody of 20 µg per mouse on day 1, 3, 5 and 7. The tumor sizes of both primary and distant tumors and body weights of the mice in different groups were measured every two days. The tumor volume was calculated according to the formula: width^2^ × length/2. The growth curves of both primary and distant tumors in each group were recorded.

### DCs Maturation Analysis of Mice in Tumors and Lymph Nodes

The left tumor tissues and the lymph nodes were obtained on day 4 after different treatments, cut into pieces, and homogenized to form single‐cell suspension by cell strainer. Cells were stained with anti‐mouse CD11c FITC and anti‐mouse CD86 PE for flow cytometry.

### Intratumoral T Cell Analysis of Mice by Flow Cytometry

Both right and left tumors were obtained from each group on the 14th day. After being cleaned with cold PBS and cut into small pieces, tumors were ground by the rubber end of a syringe in cell strainers with and incubated 0.25% trypsin‐EDTA solution for 5 min at 37 °C. Then cells were filtered using 70 µm nylon strainers to obtain suspensions of single cells. After being washed with PBS, 5% bovine serum albumin and 7‐AAD viability staining solution was added to block the unspecific protein and exclude dead cells, respectively. After that, anti‐mouse CD16/32 antibody, FITC anti‐mouse CD3 antibody, PE anti‐mouse CD4 antibody, and APC anti‐mouse CD8 antibody were added at 4 °C for 15 min in darkness to surface staining for flow cytometry. For the isotype controls, FITC anti‐mouse Rat lgG2a *κ* isotype antibody, PE/Cy7 anti‐mouse Rat lgG2a *λ* isotype antibody, PE anti‐mouse Rat lgG2a *κ* isotype antibody, FITC anti‐mouse Rat lgG2b *κ* isotype antibody, PE anti‐mouse Rat lgG2b *κ* isotype antibody, and APC anti‐mouse Rat lgG1 *κ* isotype antibody were used.

### Immunohistochemistry Analysis

The left tumors were fixed in 4% paraformaldehyde and the sections were made according to the standardized experimental procedures. After the tumors were sectioned at 4 µm thickness, CD8 or CD4 antibodies and corresponding HRP combined secondary antibodies were used. Finally, the slide was examined with a light microscope.

### Immunofluorescence Assay for T Cells

The left tumors were fixed in 4% paraformaldehyde and the sections were made according to the standardized experimental procedures. After the tumors were sectioned at 4 µm thickness, CD8 or CD4 antibodies were incubated overnight at 4 °C. After being washed with PBS twice, dye‐conjugated secondary antibodies were added at room temperature for 1 h, and a drop of anti‐fluorescence quench agent containing DAPI was dropped on the section for another 10 min. Finally, the slide was washed twice with PBS and sealed for visualizing in fluorescence microscope (Olympus; Tokyo, Japan).

### Analysis of Cytokine in the Serum of Mice

The serum of eight groups of mice was taken to test the expression of IgG, TNF‐*α*, and IFN‐*γ* according to vendors’ protocols.

### Histological Examination

The tumors on both sides of the collected animals and the major organs (heart, liver, spleen, lung, and kidney) were fixed in 4% paraformaldehyde, and the sections were made according to the standardized experimental procedures. Each organ was sectioned at 4 µm thickness and stained with H&E. The tumors on both sides of the collected animals were also used for terminal deoxynucleotidyl TUNEL staining.

### Statistical Analysis

All data were expressed as mean ± standard deviation (SD). All values were obtained from at least three independent experiments. The sample size in each group was 3 in the in vitro experiments. The sample size (*n* = 4) was for the in vivo experiments. Statistical significance was evaluated using one‐way analysis of variance (ANOVA). The difference between groups was considered statistically significant when the *p* value was lower than 0.05. Statistical significance thresholds were set at *p < 0.05; ** *p* < 0.01; and *** *p* < 0.001.

## Conflict of Interest

The authors declare no conflict of interest.

## Supporting information

Supporting Information

## Data Availability

The data that support the findings of this study are available from the corresponding author upon reasonable request.
