# Supplementary material for: Mace‐Like Plasmonic Au‐Pd Heterostructures Boost Near‐Infrared Photoimmunotherapy
Source: Adv Sci (Weinh). 2023 Jan 4;10(6):2204842. doi: 10.1002/advs.202204842 (PMC9951300; doi:10.1002/advs.202204842)
Supplement: Supplementary file 1 — Supporting Information [file ADVS-10-2204842-s001.pdf]

ADVANCED SCIENCE
Open Access

Supporting Information

for *Adv. Sci.*, DOI 10.1002/advs.202204842

Mace-Like Plasmonic Au-Pd Heterostructures Boost Near-Infrared Photoimmunotherapy

Yanlin Feng, Xin Ning, Jianlin Wang, Zhaoyang Wen, Fangfang Cao, Qing You, Jianhua Zou, Xin Zhou, Teng Sun, Jimin Cao* and Xiaoyuan Chen**

Supporting Information

Mace-like Plasmonic Au-Pd Heterostructures Boost Near-Infrared Photoimmunotherapy

Yanlin Feng[1#], Xin Ning[1#], Jianlin Wang[1], Zhaoyang Wen[1], Fangfang Cao[2,4], Qing You[2,4], Jianhua Zou[2,4], Xin Zhou[1], Teng Sun[1], Jimin Cao[1]*, Xiaoyuan Chen[2,3,4]**

[1]Key Laboratory of Cellular Physiology at Shanxi Medical University, Ministry of Education, and the Department of Physiology, Shanxi Medical University, Taiyuan 030001, China

[2]Departments of Diagnostic Radiology, Surgery, Chemical and Biomolecular Engineering, and Biomedical Engineering, Yong Loo Lin School of Medicine and Faculty of Engineering, National University of Singapore, Singapore, 119074, Singapore

[3]Clinical Imaging Research Centre, Centre for Translational Medicine, Yong Loo Lin School of Medicine, National University of Singapore, Singapore 117599, Singapore

[4]Nanomedicine Translational Research Program, NUS Center for Nanomedicine, Yong Loo Lin School of Medicine, National University of Singapore, Singapore 117597, Singapore

[5]Institute of Molecular and Cell Biology, Agency for Science, Technology, and Research (A*STAR), 61 Biopolis Drive, Proteos, Singapore, 138673, Singapore

[#] These authors contributed equally to this work.

[*] Corresponding Authors.

Email: cffdc@nus.edu.sg, caojimin@sxmu.edu.cn, chen.shawn@nus.edu.sg

Materials

Cetyltrimethylammonium bromide (CTAB, 99%), cetyltrimethylammonium chloride (CTAC, 99%), sodium tetrachloropalladium ($NaPdCl_4$), sodium oleate (NaOL), cetylpyridine chloride (CPC), sodium borohydride ($NaBH_4$) and ascorbic acid (AA) were purchased from Aladdin. Gold chloride trihydrate ($HAuCl_4 \cdot 3H_2O$, 99%) was obtained from ACMEC. Hydrochloric acid (HCl, 37 wt%), silver nitrate ($AgNO_3$, 99.8%), sodium hydroxide (NaOH, 98.2%) and sodium chloride (NaCl) were supplied by Beijing Chemical Reagent Company (China). Thiol-terminated PEG (PEG-SH, Mw = 5000) was purchased from Ponsure (Shanghai, China). Roswell Park Memorial Institute (RPMI) 1640 medium, the trypsin-EDTA, fetal bovine serum (FBS) and phosphate buffer (PBS) were all acquired from Procell. Cell Counting Kit-8 (CCK-8) was purchased from APExBIO. Lipopolysaccharide (LPS), DCFH-DA, APF, SOSG, JC-1, MitoSox Red, murine GM-CSF and murine IL-4 were acquired from Sigma. Superoxide anion Content Detection Kit was purchased from Solarbio. Celcein-AM, PI, Annexin V-FITC Apoptosis Detection Kit and One Step TUNEL Apoptosis Assay Kit were obtained from Beyotime. α-PD-L1 was acquired from Bio X Cell. Immune globulin G (IgG), tumor necrosis factor α (TNF-α) and interferon γ (IFN-γ) were purchased from BD Biosciences. Anti-CD16/32, anti-CD11c FITC, anti-CD86 PE, anti-CD80 APC, FITC antimouse CD3 antibody, PE antimouse CD4 antibody, APC antimouse CD8 antibody, 7-AAD viability staining solution, FITC antimouse Rat lgG2a κ isotype ctrl antibody, PE/Cy7 antimouse Rat lgG2a λ isotype ctrl antibody, PE antimouse Rat lgG2a κ isotype ctrl antibody, FITC antimouse Rat lgG2b κ isotype ctrl antibody, PE antimouse Rat lgG2b κ isotype ctrl antibody and APC antimouse Rat lgG1 κ isotype ctrl antibody were acquired from Biolegend. PBFI-AM was purchased from Abcam (Cambridge, United Kingdom). Pluronic F-127 was acquired from Solarbio. All of these chemicals were used without further purification. Distilled water for laboratory was obtained from the Milli-Q System.

Characterization

The morphology was characterized by Transmission electron microscopy (TEM) and STEM-high angle annular dark field (HAADF) (FEI Tecnai G20). X-ray diffraction (XRD) pattern was recorded by Rigaku-Dmax 2500 diffractometer. UV-Vis spectra were recorded with UH5700 UV-Vis/visible/NIR spectrometer. Zeta potential and dynamic light scattering (DLS) were measured by Malvern Nanosizer ZS.

Cell culture

Mouse breast cancer (4T1) cells and mouse embryonic fibroblast (NIH-3T3) cells were purchased from Shanghai Institute of Biological Sciences and cultured in a 25 cm^2 cell culture

dish using complete medium (RPMI-1640). The cell lines were placed in an incubator at 37 °C containing 5% CO_2. Cells are passed by trypsin digestion.

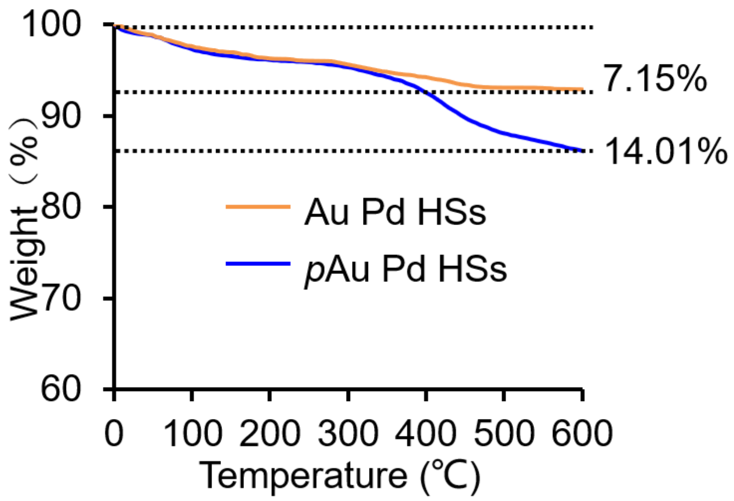

Figure S1. Thermogravimetric analysis of Au Pd HSs and *p*Au Pd HSs.

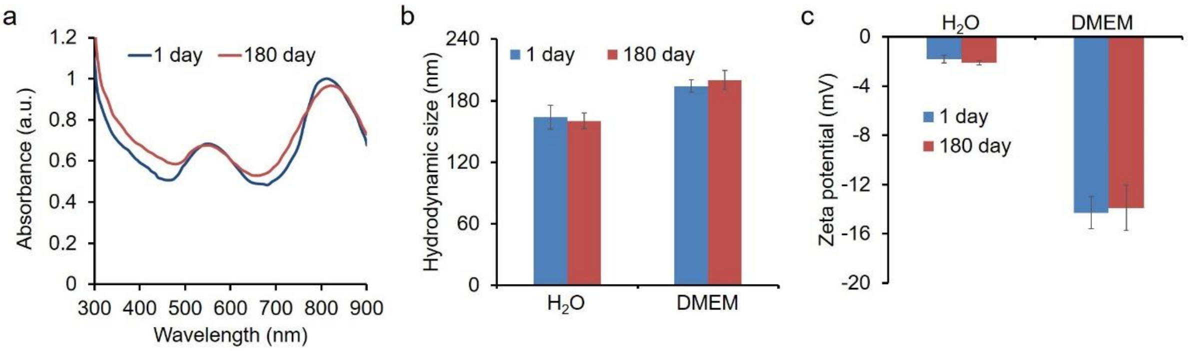

Figure S2. The storage stability of *p*Au Pd HSs for 6 month (180 days). (a) the absorption profile of *p*Au Pd HSs in DMEM medium after 1 day and 180 days storage, (b) The hydrodynamic size, and (c) zeta potential of *p*Au Pd HSs in H_2O and DMEM medium after 1 day and 180 days storage.

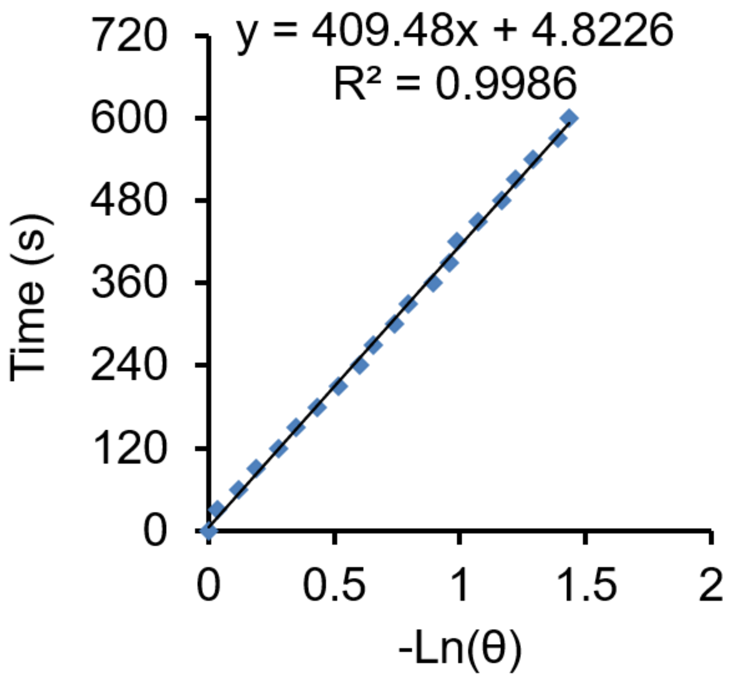

Figure S3. Plot and linear fit of time versus negative natural logarithm of the temperature elevation for the cooling rate of *p*Au Pd HSs.

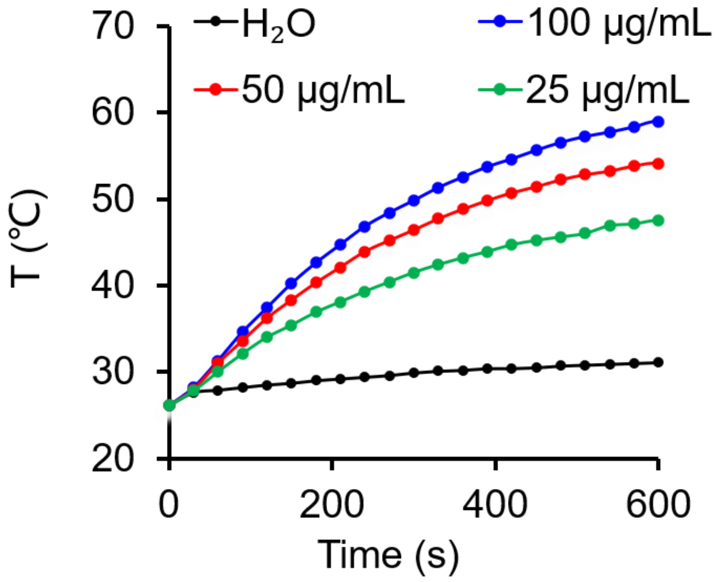

Figure S4. Temperature elevation of various concentrations of *p*Au Pd HSs with 808 nm laser irradiation (0.75 W/cm^2).

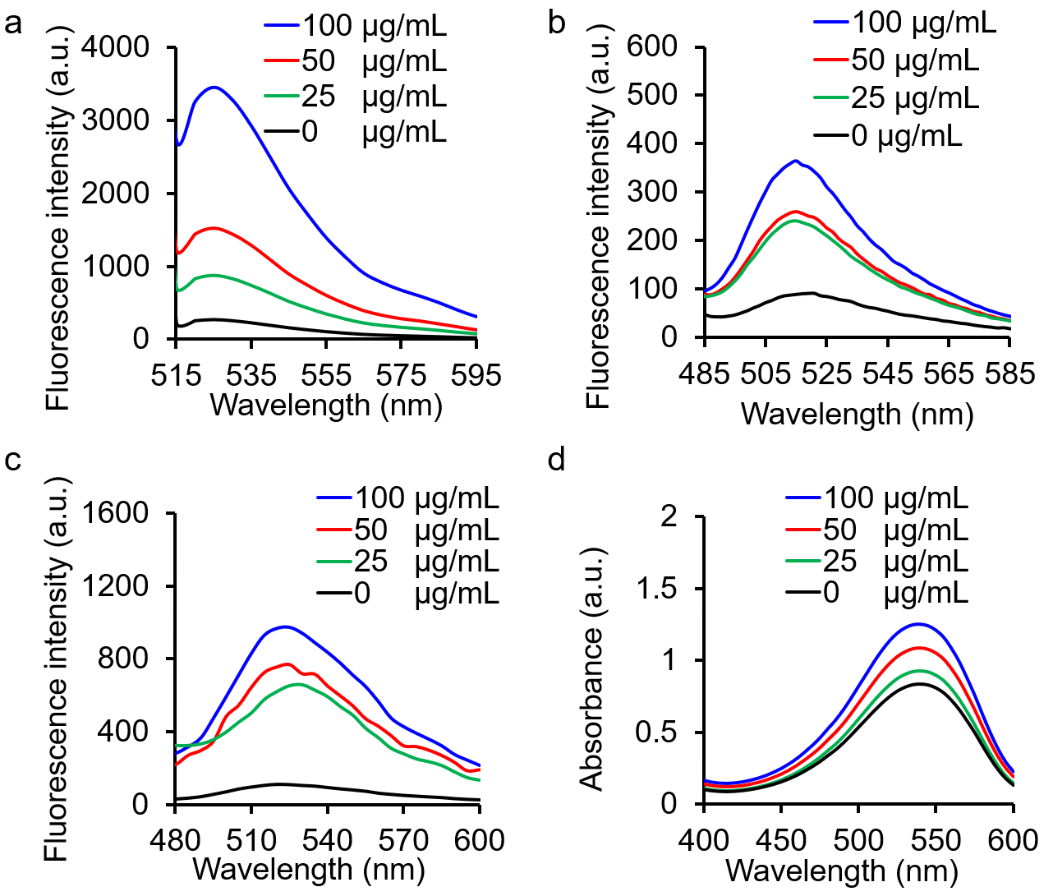

Figure S5. Total ROS, •OH, $^{1}O_2$ and O_2•$^{-}$ assessments on *p*Au Pd HSs of various concentrations with 808 nm laser irradiation (0.5 W/cm^2, 10 min) by DCF (**a**), APF (**b**), SOSG (c) and superoxide anion assay (**d**), respectively. PBS was used as control.

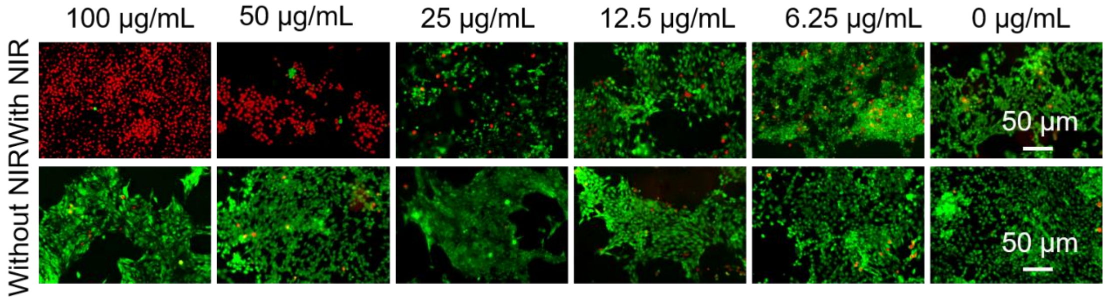

Figure S6. Live/dead staining of 4T1 cells treated with 0-100 μg/mL *p*Au Pd HSs for 24 h with or without 808 nm laser irradiation (0.5 W/cm^2, 10 min).

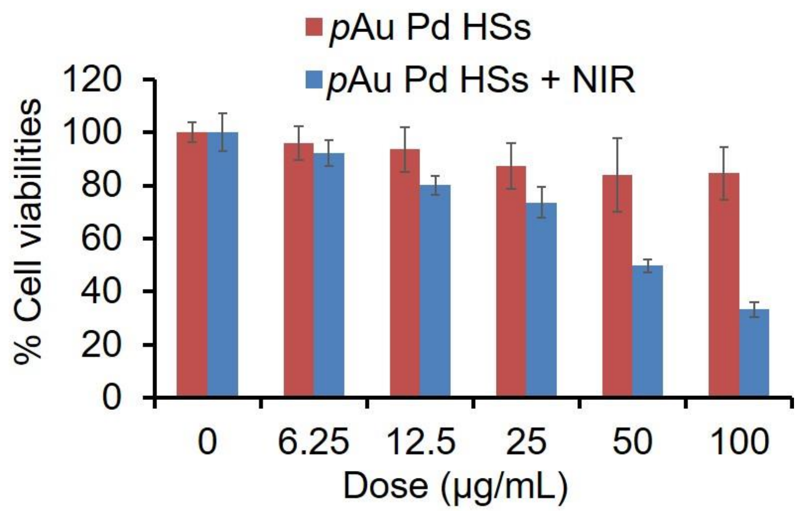

Figure S7. CCK-8-based cell viability assessment of NIH-3T3 cells after treatment of *p*Au Pd HSs at various concentrations without or with 808 nm laser irradiation.

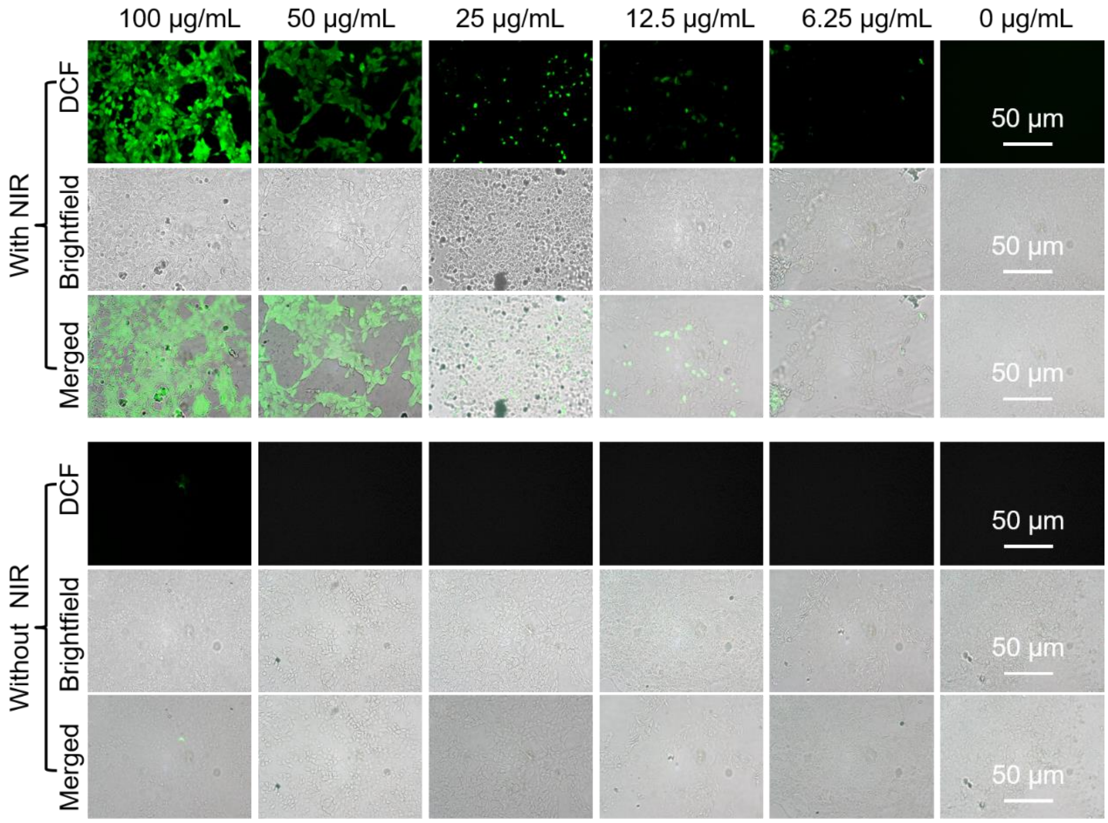

Figure S8. Fluorescence microscopy images of 4T1cells to detect intracellular ROS (DCF, green). Cell nuclei (blue) were stained with DAPI.

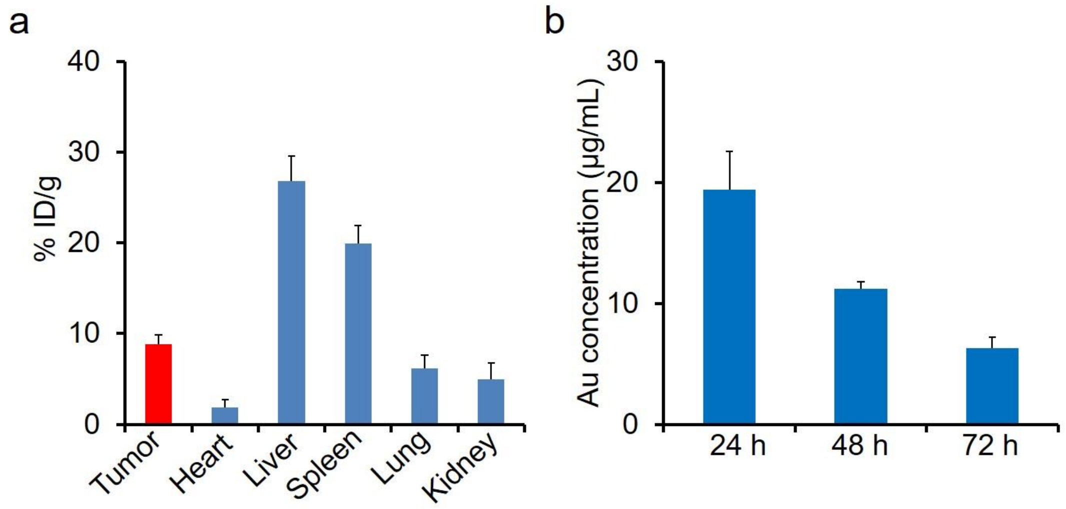

Figure S9. (a) Biodistribution of *p*Au Pd HSs in mice. (data expressed as Au percentage of the injected dose per gram of tissue (% ID/g)). (b) Au concentration in urine samples obtained at 24, 48 and 72 h after intravenously (i.v.) injection.

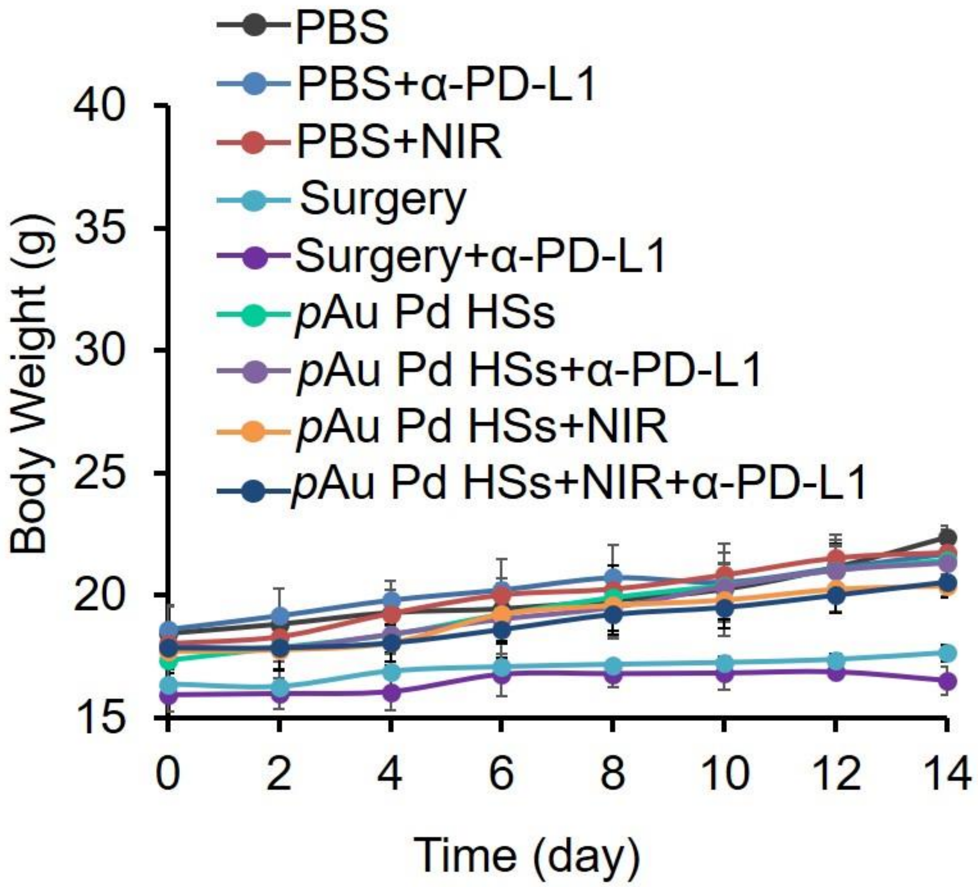

Figure S10. Body weight of mice from different groups for 14 days after various treatments.

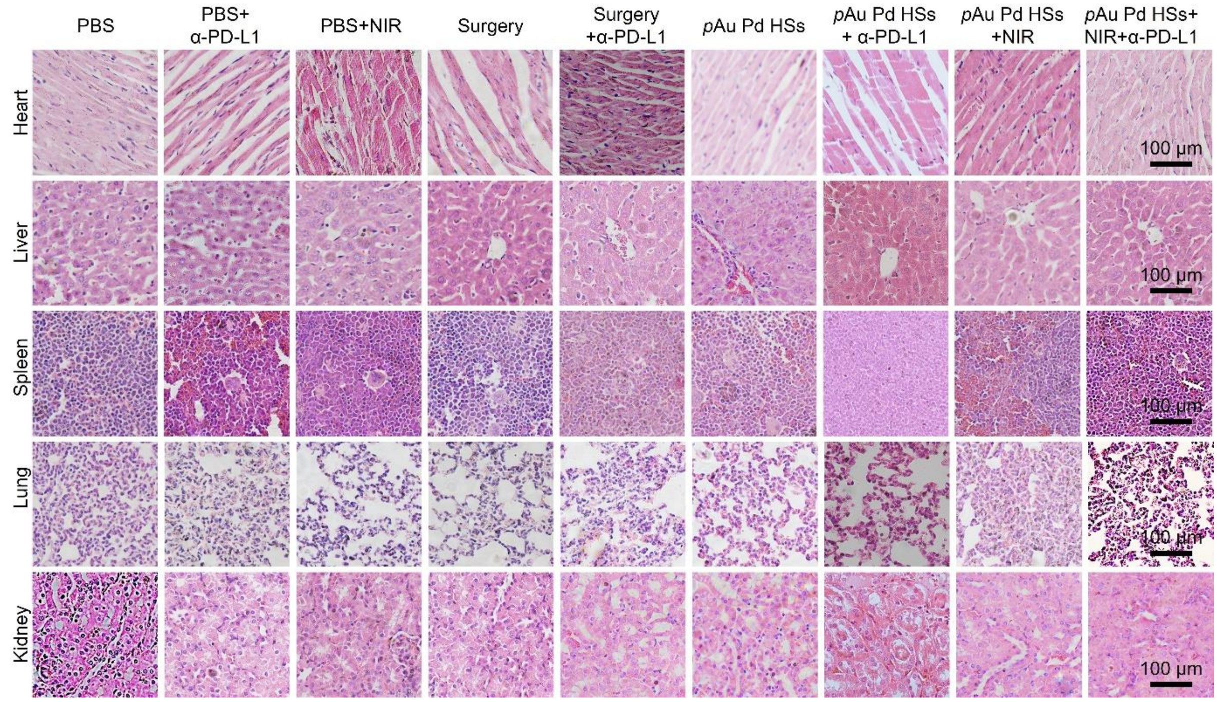

Figure S11. H&E staining histological images of heart, liver, spleen, lung, and kidney collected at the end of treatment.

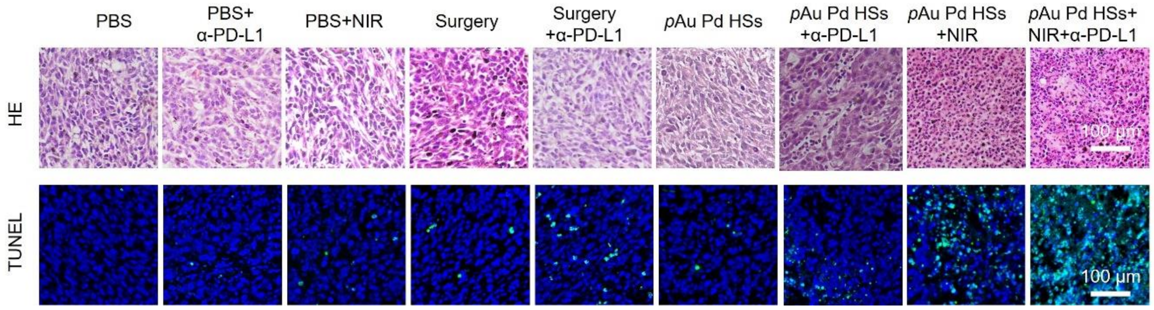

Figure S12. H&E and TUNEL staining for the left tumor tissues in each group.

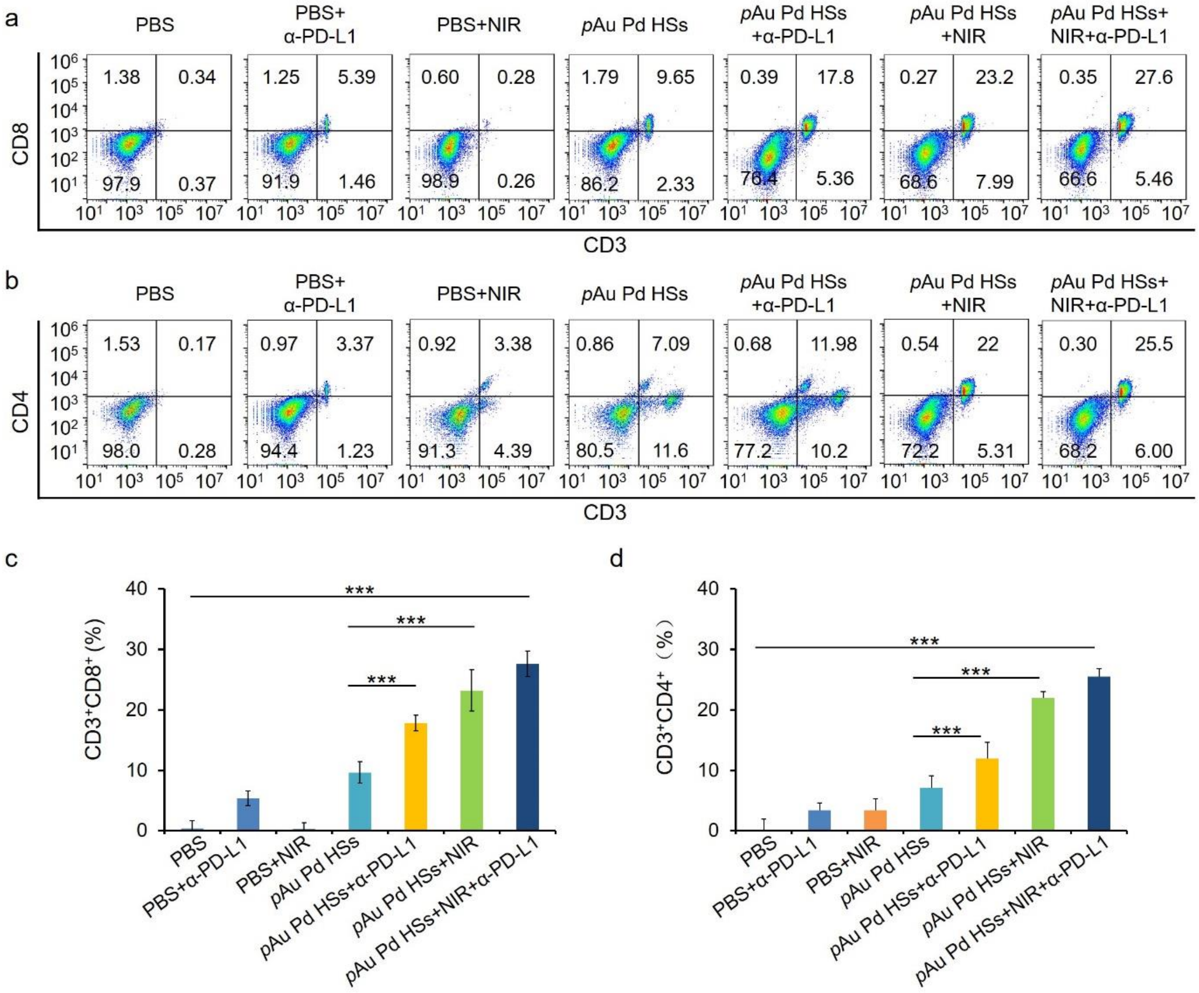

Figure S13. Typical flow cytometry plots of (a) $CD8^+$ T cells and (b) $CD4^+$ T cells in the primary tumors after different treatments. Percentages of $CD8^+$ (c) and $CD4^+$ (d) T cells with respect to the total tumor of cells.

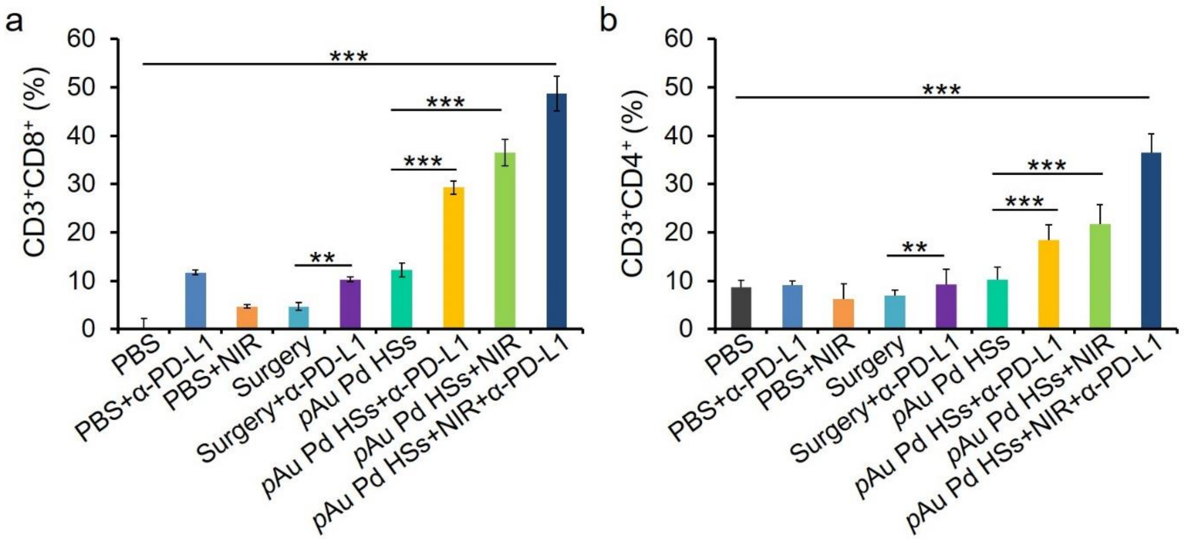

Figure S14. Percentages of $CD8^+$ (a) and $CD4^+$ (b) T cells with respect to the total tumor of cells.

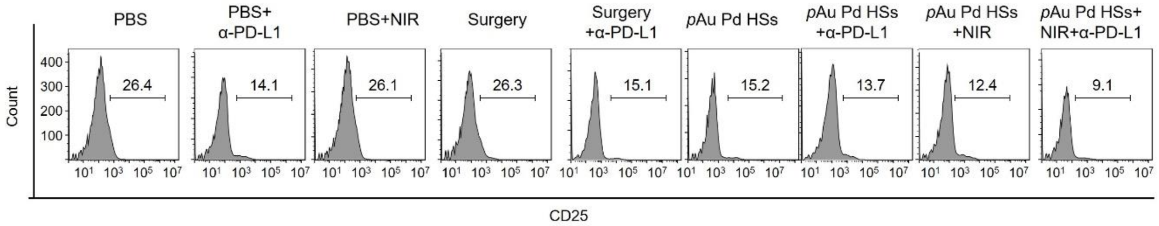

Figure S15. Flow cytometry analysis of Treg cells ($CD25^+$) in the left tumor tissues at the 14th day (gated on $CD3^+CD4^+$ cells).

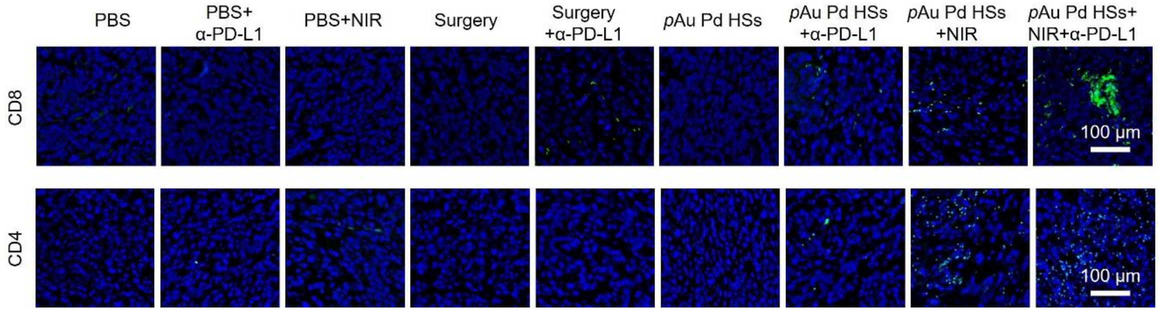

Figure S16. Immunofluorescence staining for $CD8^+$ T cells and $CD4^+$ T cells in left tumor tissues after various treatments indicated.
